# Investigation of the frequency of molar incisor hypomineralisation in childhood celiac disease and evaluation with nutritional factors and calcium metabolism

**DOI:** 10.3389/fped.2025.1603751

**Published:** 2025-08-08

**Authors:** Ayşegül Tok, Nilgün Altınsoy, Ferda Özbay Hoşnut

**Affiliations:** ^1^ Pediatric Gastroenterology and Hepatology, Etlik City Hospital, Ankara, Turkey; ^2^ Dentistry, Etlik City Hospital, Ankara, Turkey; ^3^ Gastroenterology and Hepatology, Etlik City Hospital, Ankara, Turkey

**Keywords:** celiac disease, molar incisor hypomineralisation (MIH), vitamin D, calcium metabolism, children

## Abstract

**Background:**

The relationship between celiac disease and developmental enamel defects is complex and multifaceted. Although the presence of enamel changes in individuals with celiac disease is well documented, the exact etiology of these changes remains unclear. This study aims to investigate whether the enamel defects observed in children with celiac disease are due to malabsorption-related deficiencies or are a direct consequence of the autoimmune nature of celiac disease, thus informing the development of effective preventive strategies.

**Materials and methods:**

This case-control clinical study included 150 children aged 3–18 years who were followed with a diagnosis of celiac disease, and 151 healthy controls with negative celiac serology, all evaluated at the Pediatric Gastroenterology Clinic between September 2023 and January 2025. The diagnosis of molar-incisor hypomineralization (MIH) was made based on the clinical criteria established by the European Academy of Paediatric Dentistry.

**Results:**

Celiac disease diagnosis was confirmed through positive tissue transglutaminase IgA and anti-endomysial IgA antibodies, along with histopathological findings from upper gastrointestinal endoscopy. Among the celiac patients, 36.6% were newly diagnosed, 37.3% were compliant with a gluten-free diet, and 27% were non-compliant. Molar-incisor hypomineralization (MIH) was observed in 20.7% of the children with celiac disease, compared to 6% in the healthy control group. The likelihood of MIH occurrence in children with celiac disease was found to be 8.97 times greater than in healthy controls. MIH was most prevalent among newly diagnosed and non-compliant children with celiac disease, who also exhibited significantly lower vitamin D levels and elevated tissue transglutaminase values. However, there was no significant correlation between MIH prevalence and Marsh classification of intestinal damage.

**Conclusion:**

MIH serves as a critical indicator of celiac disease, emphasizing the need for vigilant monitoring of vitamin D levels and dietary adherence to mitigate the development of MIH in affected individuals.

## Introduction

Celiac Disease (CD) is an autoimmune disease that occurs in genetically predisposed individuals after exposure to gluten for at least 6–8 months. As a result of this autoimmunity, the absorption of essential nutrients is impaired by causing histopathological damage to the small intestinal mucosa, leading to malabsorption with crypt hyperplasia, villous atrophy and inflammatory infiltration in the adjacent connective tissue, and this condition manifests clinically as diarrhoea, weight loss, fatigue, anaemia and growth retardation in children. Celiac disease is a multisystem disease. In addition to the intestines, it also affects other systems such as the heart, brain, skeleton, reproductive system, hormonal system, oral mucosa, skin and tooth enamel. Diagnosis is usually made by serological testing for specific antibodies such as anti-tissue transglutaminase and anti-endomysial antibodies, followed by a confirmatory intestinal biopsy. The basis of management is a strict lifelong gluten-free diet, which can lead to improvement of symptoms, normalisation of antibody levels and healing of the intestinal mucosa (1).

The effects of celiac disease on dental health were first observed systematically in the 1970s. Studies have gradually increased after the 2000s and Developmental Enamel Defects (DDE)s have started to be defined. Enamel defects such as pitting, grooving and discolouration have been reported in individuals with CD and have been identified as potential oral markers for this condition (2–4). Factors contributing to DDE's include immune dysregulation, nutritional disorders and genetic factors (5).

Developmental defects of enamel (DDEs) are encountered daily in clinical practice. Epidemiological data reflecting an increasing trend of this condition, with DDE prevalence in permanent dentition ranging from 10% to 49%, should be considered a public health problem and a challenge for dental practitioners (6).DDE can have a significant impact on oral health and aesthetics, tooth sensitivity and altered occlusal function (7, 8). Enamel defects are also risk conditions for dental caries and erosion in children (9, 10).

A 2015 study analysed 39 teeth from 30 children aged 13–19 using a calibrated reflectance spectrophotometer. The clinical visibility of enamel developmental defects (DDEs) is closely related to their location and optical integration with the surrounding enamel. Defects in the incisal area are more noticeable due to greater contrast in optical properties, while those in the cervical region are less perceptible. Loss of surface gloss and the presence of demarcated opacities can obscure tertiary anatomical features. Spectrophotometric analysis objectively quantifies these differences using CIE L*, a*, and b* values. Greater divergence from the optical properties of healthy enamel increases defect visibility, highlighting the importance of both localization and color contrast in clinical assessment (11).

The most commonly affected teeth are considered to be incisors and molars (5). In a meta-analysis published in 2018, a high prevalence of developmental enamel defects (DDE) was reported among individuals with celiac disease. Notably, a significant correlation was found specifically in patients undergoing primary dentition (12). Other differential diagnoses of DDE include enamel hypoplasia, fluorosis, amelogenesis imperfecta and localised trauma/infection (13, 14). Several studies have shown a high incidence of DDE in children with celiac disease, suggesting a correlation with the extent of intestinal lesions (13).

Molar incisor hypomineralization (MIH) is classified within the broader category of Developmental Defects of Enamel (DDE) and is recognized as a distinct dental phenotype with a potential association to systemic disorders such as celiac disease.

Mazur et al. reviewed the relationship between early life nutrition and the development of molar incisor hypomineralization (MIH), emphasizing that nutritional factors during pregnancy and early childhood may affect the risk of MIH, and drawing attention to appropriate maternal and infant nutrition. In addition, the study emphasizes that systemic diseases such as celiac disease may be involved in the etiology of MIH by causing nutritional deficiencies (such as calcium, vitamin D, and other minerals) that impair enamel mineralization and potentially contribute to the formation of MIH, and emphasizes the need for awareness and further research (15).

Recent systematic reviews and meta-analyses have demonstrated that children with Molar-Incisor Hypomineralization (MIH) are at a higher risk of developing dental caries compared to unaffected peers. The compromised enamel in MIH-affected teeth facilitates bacterial invasion and acid demineralization, leading to increased caries prevalence. This underscores the importance of early detection and targeted preventive interventions for children with MIH to mitigate their elevated caries risk (16).

The mechanisms underlying the formation of developmental enamel defects are hypothesized to involve hypocalcemia consequent to celiac disease, genetic predispositions such as specific HLA alleles, or autoimmune reactions (e.g., anti-amelogenin antibodies or IFN-γ/Stat-1 mediated effects) occurring during odontogenesis; however, the relationship remains debated and the precise triggers are not yet fully understood (17–20).

Studies suggested that gluten had a direct effect on enamel development and implied consequences for the mineralization process (21). The relationship between celiac disease and developmental enamel defects was complex and multifaceted. While the presence of enamel alterations in individuals with celiac disease was a well-documented fact, the exact etiology of these changes remained unclear (5, 12, 20, 21). This study aims to investigate whether the enamel defects observed in children with celiac disease result from malabsorption-related deficiencies of calcium, phosphorus, and vitamin D, or if they arise due to the autoimmune nature of celiac disease itself. Such investigations could enhance our understanding of the detrimental effects of celiac disease on dental health and contribute to the development of effective preventative strategies.

## Materials and methods

### Study design

This single-center, non-blinded, prospective case-control study was conducted at the Pediatric Gastroenterology Clinic of Ankara Etlik City Hospital, University of Health Sciences, between September 2023 and January 2025.

### Participants

A total of 150 children aged 3–18 years with celiac disease (CD) were included. Participants were either newly diagnosed or previously diagnosed based on positive tissue transglutaminase IgA and anti-endomysial IgA antibody tests, confirmed by small intestinal biopsy findings in accordance with the Marsh-Oberhuber classification:Marsh 1: Normal mucosa (low probability of CD), Marsh 2: Hyperplastic lesions (possible CD),Marsh 3: Destructive lesions (indicative of active, untreated CD).

CD patients were categorized into three subgroups:

• Newly diagnosed
• Diet-compliant (adhering to a gluten-free diet)
• Diet-noncompliant (not adhering to a gluten-free diet)

The control group comprised 151 healthy children matched for age and sex, who presented to the general pediatric outpatient clinic for routine well-child checkups or non-inflammatory conditions such as constipation, irritable bowel syndrome, or functional abdominal pain. All control subjects had negative celiac serology.

### Sample size and power analysis

Sample size determination was conducted using G*Power version 3.1.9.7. For comparisons between the CD group (*n* = 150) and the control group (*n* = 151), a two-tailed independent samples *t*-test was assumed. With an expected medium effect size (Cohen's d = 0.5), *α* = 0.05, the *post hoc* power analysis showed a statistical power of 98.9%, indicating adequate power to detect medium differences.

A second *post hoc* analysis was performed for comparisons among the three CD subgroups using one-way ANOVA. Assuming a medium effect size (Cohen's f = 0.25), *α* = 0.05, and a total of 150 participants, the achieved power was 86.2%, indicating sufficient sensitivity to detect moderate subgroup differences.

### Exclusion criteria

The following exclusion criteria were applied:

Presence of dental fluorosis
Undergoing fixed orthodontic treatment

Developmental Enamel Defects (DDEs) attributed to systemic conditions other than CD, including:

Respiratory disorders
Early childhood infectious diseases
Type 1 Diabetes
Genetic syndromes (e.g., Down syndrome, Turner syndrome)
History of premature birth
Use of any medications at the time of clinical assessment

### Oral and laboratory examinations

All participants underwent an oral examination by a single calibrated pediatric dentist. Examinations were conducted with a mouth mirror and blunt dental probe under artificial lighting in the Pedodontics Clinic, with the patient seated in a dental chair.

The diagnosis of Molar Incisor Hypomineralization (MIH) followed the European Academy of Paediatric Dentistry (EAPD) criteria (6). MIH was recorded when at least one first permanent molar displayed one or more of the following:

Demarcated opacities
Atypical restorations
Post-eruptive enamel breakdown (PEB)
Extraction due to MIH-related complications

Laboratory data including serum calcium, phosphorus, magnesium, vitamin D, and parathyroid hormone (PTH) levels were obtained from routine follow-up records.

### Statistical analysis

All statistical analyses were conducted using IBM SPSS Statistics version 23.0.Descriptive data were presented as mean ± standard deviation, median (interquartile range), or frequency (percentage) as appropriate.Comparisons were made using:Chi-square test or Fisher's exact test for categorical variables, Student's t-test or Mann–Whitney *U* test for continuous variables,For comparisons across Marsh types, the Kruskal–Wallis test was applied. A *P*-value < 0.05 was considered statistically significant.

### Ethical considerations

This study was approved by the Ethics Committee of Etlik City Hospital, Ankara (Approval No: AESH-EK1-2023-363, Date: 09/06/2023). The study was conducted in accordance with the principles outlined in the Declaration of Helsinki and its amendments. Informed written consent was obtained from all participants and/or their legal guardians prior to inclusion in the study.

## Results

### Demographic data

The mean age of 150 (102 girls/48 boys) patients in the celiac patient group was 9.76 ± 4.03 years (range 3–18 years); the mean age of 151 (78 girls/73 boys) patients in the control group was 11.59 ± 3.93 years. There was a significant difference between the two groups in terms of gender. (*p* < 0.05).

The rate of MIH in children with celiac disease was 20.7%, while this rate was 6% in the healthy control group (<0.001) (Table 1). In addition, our study found that celiac disease increased the probability of MIH by 8.9 times (95% Confidence Interval 8.976 ± 1.882). The location of enamel defects in permanent and primary teeth was more pronounced in the anterior teeth, and the coronal distribution included the incisors and the central parts of the teeth.

**Table 1:** MIH positivity rates in children with celiac disease group and control group.

	Children with celiac disease group (*n* = 150) *n* (%)	Control group (*n* = 151) *n* (%)	*p*
MIH (+)	31 (20.7%)	9 (6.0%)	<0.001
MIH(−)	119 (79.3%)	142 (94.0%)	

There was a significant difference between the children with celiac disease group and the control group in terms of the mean magnesium, parathyroid hormone and vitamin D levels measured in routine controls of all these patients (Table 2).

**Table 2:** Laboratory findings in children with celiac disease group and control group.

	Children with celiac disease group (*n* = 150) *n* (%)	Control group (*n* = 151) *n* (%)	*p*
Vitamin D (20–40 ng/ml)	17.82 ± 7.72	19.23 ± 6.60	**0** **.** **024** **
Calcium (8.8–10.8 mg/dl)	9.47 ± 1.02	10.41 ± 7.69	0.217
Phosphorus (4.5–5.5 mg/dl)	4.68 ± 0.83	4.56 ± 80.75	0.804
Magnesium (1.6–2.6 mg/dl)	2.52 ± 3.32	2.51 ± **0.**31	0.529
Parathyroid hormone (11.3–60 pg/ml)	45.56 ± 67.31	34.99 ± 12.8	**0**.035**

Data are presented as mean ± standard deviation (SD). Comparisons between groups were performed using the independent samples *t*-test. A *p*-value of <0.05 was considered statistically significant. Statistically significant values are indicated in bold (**).

In the celiac patient group, there were 55 patients diagnosed for the first time (36.7%), 56 patients compliant with the diet for the last year (37.3%) and 39 patients not compliant with the diet for the last year (26%). The MIH incidence rates of these patients, the mean tissue TGA levels, calcium, phosphorus, magnesium, parathyroid hormone and vitamin D levels measured in routine controls are given in Table 3.

**Table 3:** Comparison of diet compliance with laboratory parameters and tissue TGA and MIH presence in children with celiac disease group.

Children with celiac disease group (*n* = 150)	New diagnosis (*n* = 55)	Diet compliant (*n* = 56)	Diet noncompliant (*n* = 39)	*p*
Vitamin D (20–40 ng/ml)	18.76 ± 8.47	18.72 ± 6.78	**15.20** **±** **7.47**	**0**.046**
Calcium (8.8–10.8 mg/dl)	9.64 ± 0.46	9.37 ± 1.46	9.37 ± 0.78	0.070
Phosphorus (4.5–5.5 mg/dl)	4.90 ± 0.67	4.06 ± 0.82	4.48 ± 0.99	0.151
Magnesium (1.6–2.6 mg/dl)	2.29 ± 1.16	3.17 ± 5.41	1.99 ± 0.37	0.052
Parathyroid hormone (11.3–60 pg/ml)	40.48 ± 20.74	47.94 ± 101.28	49.29 ± 51.34	0.240
tTG IgA (IU/ml)^a^	135.41 ± 75.99	13.64 ± 40.06	81.17 ± 74.87	<0.001**
MIH(n = 31)	17	6	8	0.029**

Data are presented as mean ± standard deviation (SD). Group comparisons were performed using one-way ANOVA for continuous variables and chi-square test for categorical variables (e.g., MIH prevalence). A *p*-value <0.05 was considered statistically significant. Statistically significant values are indicated in bold (**).

Vitamin D and tissue transglutaminase levels were significantly different in patients with MIH (+).

^a^ Tissue transglutaminase(tTG IgA) (IU/ml): negative <12, border = 12–18, positve >18.

**Table 4:** Comparison of calcium metabolism parameters between celiac children with and without MIH.

	CD with MIH	CD without MIH	*p*
Vitamin D (20–40 ng/ml)	13.75 ± 6.97	17.84 ± 7.66	**0**.008**
Calcium (8.8–10.8 mg/dl)	9.57 ± 0.55	9.44 ± 1.11	0.070
Phosphorus (4.5–5.5 mg/dl)	4.79 ± 0.58	4.66 ± 0.88	0.541
Magnesium (1.6–2.6 mg/dl)	2.32 ± 1.56	2.58 ± 3.67	0.978
Parathyroid hormone (11.3–60 pg/ml)	42.91 ± 32.59	46.29 ± 74.15	0.905
tTG IgA (IU/ml)^a^	114.80 ± 85.77	65.70 ± 79.51	**0**.006**

CD, celiac disease; MIH, molar incisor hypomineralization; tTG IgA, tissue transglutaminase IgA. Data are presented as mean ± standard deviation (SD). Comparisons were made using the independent samples *t*-test. A *p*-value <0.05 was considered statistically significant. Statistically significant values are shown in bold (**).

^a^ Tissue transglutaminase(tTG IgA) (IU/ml): negative <12, border = 12–18, positve >18.

Vitamin D levels were significantly lower in the diet-adherent cases than in the other two groups. Vitamin D levels were significantly lower in patients with MIH than in those without. At the same time, tissue transglutaminase (tTG IgA) levels were significantly higher in patients with MIH than in those without (Table 4).

According to the Marsh Classification, which is the Pathological Classification of Children with celiac disease, no statistical difference was found between the rates of MIH (Table 5).

**Table 5:** Comparison of MIH patients with the Marsh classification for Celiac Disease.

	Marsh 2 (*n* = 51)	Marsh 3a (*n* = 35)	Marsh 3b (*n* = 38)	Marsh 3c (*n* = 26)	Total (*n* = 150)	*p*
Patients of MIH n (%)	9(%21.4)	5(%16.6)	8(%26.6)	9(%52.9)	31(%20.6)	0.240
Patients without MIH(−) n (%)	42(%78.6)	30(%83.4)	30(%73.4)	17(%47.1)	119(%79.4)	

MIH, molar incisor Hypomineralization. Data are presented as number of patients and column percentage (%). Statistical analysis was performed using the chi-square test.

Table 5 shows that while the MIH rate was higher in type 3c (Marsh classification), the difference was not statistically significant (*p* > 0.05).

## Discussion

Molar incisor hypomineralization (MIH) is a significant health problem that can affect the child's quality of life by negatively affecting their esthetics and function.

Regarding aetiology, perinatal hypoxia, prematurity and other hypoxia related perinatal problems, including caesarean section, appear to increase the risk of having MIH, while certain infant and childhood illnesses are also linked with MIH (22). In addition, genetic predisposition and the role of epigenetic influences are becoming clearer following twin studies and genome and single-nucleotide polymorphisms analyses in patients and families. Missing genetic information might be the final key to truly understand MIH aetiology (22).

In recent years, the worldwide prevalence of MIH has been reported to be between 2.9% and 44% (23, 24). In our study, the prevalence of MIH in the healthy control group was 6%.

The prevalence of celiac disease has been increasing in recent years (25). Therefore, many clinical manifestations of celiac disease are becoming more prominent. The prevalence of MIH was 61% in Çiğdem Elbek-Çubukçu's study (26), 66.9% in Ahmed A's study (14), 42.2% in Avşar and Kalaycı's (27), 40% in Acar et al. (28), reported a higher prevalence of enamel defects in children with CD than in controls; and respectively. Kuklik's study (29) also aimed to analyse the incidence of MIH in patients with CD compared with the CG. Among participants with CD, 20 percent had MIH, while among those without the disease, only 5 percent showed the condition. This difference was statistically significant (*p* = 0.044), indicating an association between CD and the occurrence of MIH. Most of the defects in patients with CD consisted of demarcated opacities. In our study, similar to this study, 20% of the participants with CD had MIH, whereas only 6% of those without CD had MIH. Kuklik et al. reported that the probability of MIH in children with CD is 4.75 times Ahmed A. et al. 8.1 times; higher than in controls (14, 29). In our study, we found that the probability of MIH in children with celiac disease is 8.97 times higher than in healthy controls. According to our findings, the rate of MIH was found to be 20.7% in the celiac patient group.

A predominance of female participants in children with celiac disease group resulted in a significant sex distribution difference between the patient and control groups. This observation aligns with the findings of a comprehensive systematic review and meta-analysis by Jansson-Knodell et al., which demonstrated a 42% increased risk of undiagnosed celiac disease in females compared to males, potentially reflecting underlying immunological and hormonal factors as well as differences in healthcare utilization (30).

When the nutritional values of the celiac and control groups were compared, statistically significant lower values were observed in vitamin D and parathormone levels in the celiac group (Table 2). This was expected in our patient group which is a mixed group in terms of dietary compliance (31). Vitamin D levels were lower in paediatric patients with CD compared with healthy controls (32).

When we compared the nutritional values within the celiac group to better evaluate the low nutritional values in children with celiac disease, we observed that vitamin D was statistically significantly lower especially in diet-incompliant patients. In addition, the rate of MIH was higher in diet-incompliant patients 8/39 (20%) and in newly diagnosed patients 17/55 (30.9%) compared to diet-compliant patients 8/56 (14.2%). Poor intestinal absorption of some nutrients due to celiac disease is thought to cause enamel defects (32). In a 2017 study, it was observed that there was a significant relationship between the age of starting gluten-free diet and Molar Incisor Hypommineralisation (MIH) in children with celiac disease (5). With respect to MIH, results are conflicting (5). In a German study, a positive relationship between lower vitamin D (25(OH)D) levels and increased prevalence of MIH was determined (33); however, van der Tas and colleagues reported no link between vitamin D levels at 6 years of age with MIH or hypomineralized second primary molars(HSPM) in Dutch children (34). In a recent study, a link with higher levels of vitamin D and HSPM was reported; however, the authors stated that caution should be taken when interpreting the results as they could be influenced by unknown confounding factors (29). Furthermore, a 10 nmol/L increase in serum 25(OH)D concentrations was significantly associated with a lower likelihood of having MIH (OR = 0.89; *P* = 0.006). Moreover, higher 25(OH)D values were associated with fewer caries-affected permanent teeth (29).

The findings of Nørrisgaard et al.'s randomized clinical trial provide compelling evidence for the modulatory role of prenatal high-dose vitamin D supplementation in reducing the incidence of enamel defects, including Molar Incisor Hypomineralization (MIH), in offspring (35). Given the pivotal function of vitamin D in calcium homeostasis and amelogenesis, their results suggest that adequate maternal vitamin D status during critical periods of tooth development may mitigate hypomineralization processes (35). This aligns with the hypothesis that nutritional and metabolic factors during gestation exert a significant influence on enamel quality, potentially modulating susceptibility to MIH (35). Consequently, these findings underscore the importance of optimizing prenatal vitamin D levels as a preventive strategy against developmental enamel defects, warranting further investigation into the mechanistic pathways linking vitamin D metabolism and enamel biomineralization (35).

In our study, when the nutritional parameters of children with celiac disease with MIH were compared with those without MIH, low vitamin D levels as well as high tissue transglutaminase A levels were statistically significant. There was no study in the literature on this subject with a sufficient number of patients. In our study, the significant elevation of Tissue Transglutaminase A in MIH positive patients and the fact that the majority of MIH positive patients were newly diagnosed patients and non-compliant to diet indicate an increase in autoinflammation. Especially proinflammatory cytokines, parathyroid function abnormalities and mostly nuclear factor B receptor activator/nuclear factor B receptor activator-ligand/osteoprotegerin system disorders are thought to cause bone damage (36, 37).

Our results showed that there was no significant correlation between the MIH variable and Marsh types in children with celiac disease. Although the incidence of MIH is high in Marsh type 3c, no statistically significant difference was observed (Table 5). In another study conducted in our country including 62 children with celiac disease, there was also no significant correlation between MIH and Marsh classification. Only in children with Marsh 2 scores, a higher number of dental careers in permanent teeth was reported (26).

## Conclusion

Our study is one of the studies with the highest number of cases in the literature. Celiac disease increases the incidence of MIH 8.9 times. In our study, the incidence of MIH was 6% in healthy children and 20.7% in children diagnosed with celiac disease. Vitamin D deficiency was low in all children with celiac disease, especially in diet non-compliant patients, but it was significantly low in children with celiac disease with MIH. These findings suggest that vitamin D deficiency due to malabsorption and unstoppable inflammation are important in the pathogenesis of MIH in celiac disease. MIH is an important indicator of celiac disease and attention should be paid to vitamin D levels and dietary compliance to prevent the development of MIH in patients with celiac disease.

## Data Availability

The datasets presented in this study can be found in online repositories. The names of the repository/repositories and accession number(s) can be found in the article/Supplementary Material.

## References

[1] Corazza GR Troncone R Lenti MV Silano M. Pediatric and Adult Celiac Disease: A Clinically Oriented Perspective. 1st Eds. Amsterdam: Elsevier (2024). 10.1016/B978-0-443-13359-6.00001-0

[2] Popp A Mäki M. Gluten-induced extra-intestinal manifestations in potential celiac disease-celiac trait. Nutrients. (2019) 11:320. 10.3390/nu11020320 30717318 PMC6412544

[3] Bulut M Tokuc M Aydin MN Civan HA Polat E Dogan G Advancing dentistry: fractal assessment of bone health in pediatric patients with celiac disease using dental images. Quintessence Int. (2023) 54:822–31. 10.3290/j.qi.b4325347 37602781

[4] Al-Zahrani MS Alhassani AA Zawawi KH. Clinical manifestations of gastrointestinal diseases in the oral cavity. Saudi Dent. J. (2021) 33:835–41. 10.1016/j.sdentj.2021.09.017 34938023 PMC8665164

[5] de Queiroz AM Arid J de Carvalho FK da Silva AB Küchler C Sawamura R Assessing the proposed association between DED and gluten-free diet introduction in celiac children. Spec Care Dentist. (2017) 37:194–8. 10.1111/scd.12227 28677884

[6] A review of the developmental defects of enamel index (DDE index). commission on oral health, research and epidemiology. Report of an FDI working group. Int Dent J. (1992) 42(6):411–26. 1286924

[7] Seow WK Ford D Kazoullis S Newman B Holcombe T. Comparison of enamel defects in the primary and permanent dentitions of children from a low-fluoride district in Australia. Pediatr Dent. (2011) 33(3):207–12. 21703072

[8] Jälevik B Klingberg GA. Dental treatment, dental fear and behaviour management problems in children with severe enamel hypomineralization of their permanent first molars. Int J Paediatr Dent. (2002) 12(1):24–32. 10.1046/j.0960-7439 11853245

[9] Kazoullis S Seow WK Holcombe T Newman B Ford D. Common dental conditions associated with dental erosion in schoolchildren in Australia. Pediatr Dent. (2007) 29(1):33–9. 18041510

[10] Hong L Levy SM Warren JJ Broffitt B. Association between enamel hypoplasia and dental caries in primary second molars: a cohort study. Caries Res. (2009) 43(5):345–53. 10.1159/000231571 19648745 PMC2814013

[11] Guerra F Mazur M Corridore D Pasqualotto D Nardi GM Ottolenghi L. Evaluation of the esthetic properties of developmental defects of enamel: a spectrophotometric clinical study. ScientificWorldJournal. (2015) 2015:878235. 10.1155/2015/878235 25874260 PMC4385663

[12] Souto-Souza D da Consolação Soares ME Rezende VS de Lacerda Dantas PC Galvão EL Falci SGM. Association between developmental defects of enamel and celiac disease: a meta-analysis. Arch Oral Biol. (2018) 87:180–90. 10.1016/j.archoralbio.2017.12.025 29306074

[13] Trotta L Biagi F Bianchi PI Marchese A Vattiato C Balduzzi D Developmental enamel defects (DDE)s in adult coeliac disease: prevalence and correlation with symptoms and age at diagnosis. Eur J Intern Med. (2013) 24(8):832–4. 10.1016/j.ejim.2013.03.007 23571066

[14] Ahmed A Singh A Kajal S Chauhan A Rajput MS Banyal V Developmental enamel defects (DDE)s and oral cavity manifestations in Asian patients with celiac disease. Indian J Gastroenterol. (2021) 40:402–9. 10.1007/s12664-021-01175-7 34244963

[15] Mazur M Corridore D Jedlinski M Ndokaj A Straker M Guerra F. Diet during pregnancy and early life and molar incisor hypomineralization: a systematic review. Int J Child Health Nutr. (2023) 12(4):120–8. 10.6000/1929-4247.2023.12.04.1

[16] Mazur M Corridore D Ndokaj A Ardan R Vozza I Babajko S MIH and dental caries in children: a systematic review and meta-analysis. Healthcare (Basel). (2023) 11(12):1795. 10.3390/healthcare11121795 37372913 PMC10298042

[17] Wierink CD van Diermen DE Aartman IH Heymans HS. Dental enamel defects in children with coeliac disease. Int J Paediatr Dent. (2007) 17(3):163–8. 10.1111/j.1365-263X.2006.00816.x 17397459

[18] El-Hodhod MA El-Agouza IA Abdel-Al H Kabil NS Bayomi KA. Screening for celiac disease in children with dental enamel defects. ISRN Pediatr. (2012) 2012:763783. 10.5402/2012/763783 22720168 PMC3376764

[19] Yotsumoto K Sanui T Tanaka U Yamato H Alshargabi R Shinjo T Amelogenin downregulates interferon gamma-induced major histocompatibility complex class II expression through suppression of euchromatin formation in the class II transactivator promoter IV region in macrophages. Front Immunol. (2020) 11:709. 10.3389/fimmu.2020.00709 32373130 PMC7186442

[20] de Carvalho FK de Queiroz AM Bezerra da Silva RA Sawamura R Bachmann L Bezerra da Silva LA Oral aspects in celiac disease children: clinical and dental enamel chemical evaluation. Oral Surg Oral Med Oral Pathol Oral Radiol. (2015) 119:636–43. 10.1016/j.oooo.2015.02.483 25840513

[21] Paul SP Kirkham EN John R Staines K Basude D. Coeliac disease in children—an update for general dental practitioners. Br Dent J. (2016) 220(9):481–5. 10.1038/sj.bdj.2016.336 27173708

[22] Lygidakis NA Garot E Somani C Taylor GD Rouas P Wong FSL. Best clinical practice guidance for clinicians dealing with children presenting with molar-incisor-hypomineralisation (MIH): an updated European academy of paediatric dentistry policy document. Eur Arch Paediatr Dent. (2022) 23(1):3–21. 10.1007/s40368-021-00668-5 34669177 PMC8926988

[23] Elfrink MEC Ghanim A Manton DJ Weerheijm KL. Standardised studies on molar incisor hypomineralisation (MIH) and hypomineralised second primary molars (HSPM): a need. Eur Arch Paediatr Dent. (2015) 16:247–55. 10.1007/s40368-015-0179-7 25894247

[24] Schwendicke F Elhennawy K Krois J. Prevalence, incidence, and burden of molar incisor hypomineralization. In: Bekes K, editor. Molar Incisor Hypomineralization. Vienna: Springer (2020). p. 21–31.

[25] Sahin Y. Celiac disease in children: a review of the literature. World J Clin Pediatr. (2021) 10(4):53–71. 10.5409/wjcp.v10.i4.53 34316439 PMC8290992

[26] Elbek-Cubukcu C Arsoy H-A Ozkaya G. Assessment of oral manifestations in pediatric patients with celiac disease in relation to marsh types. Med Oral Patol Oral Cir Bucal. (2023) 28:e9–e15. 10.4317/medoral.25490 36565215 PMC9805338

[27] Avşar A Kalayci AG. The presence and distribution of developmental enamel defects (DDE)s and caries in children with celiac disease. Turk J Pediatr. (2008) 50:45–50. 18365591

[28] Acar S Yetkıner AA Ersın N Oncag O Aydogdu S Arıkan C. Oral findings and salivary parameters in children with celiac disease: a preliminary study. Med Princ Pract. (2012) 21(2):129–33. 10.1159/000331794 22024774

[29] Kuklik HH Cruz ITSA Celli A Fraiz FC da AssunÇÃo LRS. Molar incisor hypomineralization and celiac disease. Arq Gastroenterol. (2020) 57:167–71. 10.1590/s0004-2803.202000000-31 32490904

[30] Jansson-Knodell CL Hujoel IA West CP Taneja V Prokop LJ Rubio-Tapia A Sex difference in celiac disease in undiagnosed populations: a systematic review and meta-analysis. Clin Gastroenterol Hepatol. (2019) 17(10):1954–1968.e13. 10.1016/j.cgh.2018.11.013 30448593

[31] Wierdsma NJ Berkenpas M Mulder CJJ Van Bodegraven AA. Vitamin and mineral deficiencies are highly prevalent in newly diagnosed celiac disease patients. Nutrients. (2013) 5:3975–92. 10.3390/nu5103975 24084055 PMC3820055

[32] Sun Y Zhou Q Tian D Zhou J Dong S. Relationship between vitamin D levels and pediatric celiac disease: a systematic review and meta-analysis. BMC Pediatr. (2024) 24(1):185. 10.1186/s12887-024-04688-0 38491474 PMC10943820

[33] Kuhnisch J Thiering E Kratzsch J Heinrich-Weltzien R Hickel R Heinrich J Elevated serum 25(OH)-vitamin D levels are negatively correlated with molar-incisor hypomineralization. J Dent Res. (2015) 94(2):381–7. 10.1177/0022034514561657 25503610 PMC4438736

[34] van der Tas JT Elfrink MEC Heijboer AC Rivadeneira F Jaddoe VWV Tiemeier H Foetal, neonatal and child vitamin D status and enamel hypomineralization. Community Dent Oral Epidemiol. (2018) 46(4):343–51. 10.1111/cdoe.12372 29493792 PMC6446811

[35] Nørrisgaard PE Haubek D Kühnisch J Chawes BL Stokholm J Bønnelykke K Association of high-dose vitamin D supplementation during pregnancy with the risk of enamel defects in offspring: a 6-year follow-up of a randomized clinical trial. JAMA Pediatr. (2019) 173(10):924–30. 10.1001/jamapediatrics.2019.2545 31381020 PMC6686764

[36] Garrote JA Gómez-González E Bernardo D Arranz E Chirdo F. Celiac disease pathogenesis: the proinflammatory cytokine network. J Pediatr Gastroenterol Nutr. (2008) 47(Suppl 1):S27–32. 10.1097/MPG.0b013e3181818fb9 18667914

[37] Larussa T Suraci E Nazionale I Abenavoli L Imeneo M Luzza F. Bone mineralization in celiac disease. Gastroenterol Res Pract. (2012) 2012:198025. 10.1155/2012/198025 22737164 PMC3378976

